# MicroCore: Mapping Genome Expression to Cell Pathways and Networks

**DOI:** 10.1002/cfg.362

**Published:** 2004-02

**Authors:** Manish Patel, Sylvia Nagl

**Affiliations:** 1 Department of Clinical Oncology Royal Free and University College Medical School Rowland Hill Street London NW3 2PF UK; 2 Department of Biochemistry and Molecular Biology University College London Gower Street London WC1E 6BT UK

## Abstract

The MicroCore toolkit is a suite of analysis programs for microarray and proteomics data that is open source and programmed exclusively in Java. MicroCore provides a flexible and extensible environment for the interpretation of functional genomics data through visualization. The first version of the application (downloadable from the MicroCore website: http://www.ucl.ac.uk/oncology/MicroCore/microcore.htm), implements two programs—PIMs (protein interaction maps) and MicroExpress—and is soon to be followed by an extended version which will also feature a fuzzy k-means clustering application and a Java-based R plug-in for microarray analysis. PIMs and MicroExpress provide a simple yet powerful way of graphically relating large quantities of expression data from multiple experiments to cellular pathways and biological processes in a statistically meaningful way.


**Comparative and Functional Genomics**
*Comp Funct Genom* 2004; **5**: 75–78.
Published online in Wiley InterScience (www.interscience.wiley.com). **DOI:** 10.1002/cfg.362

**Conference Review**

### MicroCore: mapping genome expression to cell pathways and networks

Manish Patel[1] and Sylvia Nagl[1,2]*

[1] *Department of Clinical Oncology, Royal Free and University College Medical School, Rowland Hill Street, London NW3 2PF, UK*
[2] *Department of Biochemistry and Molecular Biology, University College London, Gower Street, London WC1E 6BT, UK*

**Correspondence to: Sylvia Nagl, Department of Clinical Oncology, Royal Free and University College Medical School, Rowland Hill Street, London NW3 2PF, UK. E-mail: nagl@biochem.ucl.ac.uk*

Received: 13 November 2003
Revised: 18 November 2003
Accepted: 25 November 2003

### Abstract

**The MicroCore toolkit is a suite of analysis programs for microarray and proteomics data that is open source and programmed exclusively in Java. MicroCore provides a flexible and extensible environment for the interpretation of functional genomics data through visualization. The first version of the application (downloadable from the MicroCore website: http://www.ucl.ac.uk/oncology/MicroCore/microcore.htm), implements two programs — PIMs (protein interaction maps) and MicroExpress — and is soon to be followed by an extended version which will also feature a fuzzy k-means clustering application and a Java-based R plug-in for microarray analysis. PIMs and MicroExpress provide a simple yet powerful way of graphically relating large quantities of expression data from multiple experiments to cellular pathways and biological processes in a statistically meaningful way. Copyright © 2004 John Wiley & Sons, Ltd.**

**Keywords:** **bioinformatics; pathway analysis; genome expression; functional genomics data visualization**

### Introduction

The successful sequencing of the human genome, together with other whole-genome sequencing projects [6], have brought about a shift in research focus, marked by a transition from a sequence-orientated to a more gene-functional approach. New high-throughput methods for large-scale analysis of genome expression are enabling the generation of very large amounts of data, and the challenge now is to exploit this data in ways that enhance our understanding of biological processes and the integrated functioning of the cell.

Microarrays are a new technology for quantifying the relative amounts of RNA in a cell sample and thereby establishing an approximation of cellular gene expression at the time of RNA extraction [9]. By performing microarray analysis comparing distinct cell types, or a single cell type under a wide range of conditions, one can obtain what is commonly termed a *gene expression profile* for each cell type or experimental condition. For example, Ross *et al.* [10] and Scherf *et al.* [11] have shown that the NCI60 cancer cell lines have very specific gene expression patterns and, further, that their expression profiles correlate with drug sensitivity and resistance.

Microarray data analysis commonly applies a range of methods, ranging from hierarchical clustering and k-means clustering to neural networks and support vector machines for the identification of co-expressed genes (for recent reviews, see e.g. [15] and [12]); and the aforementioned NCI60 experiments are an example that clearly shows the power of using such techniques. Since Eisen's application of hierarchical clustering [5], this kind of analysis has become something of a paradigm to microarray data miners. Open source software packages, combining clustering techniques with data normalization and statistical analysis tools, have been released, such as Genesis [13], BioConductor, TM4 and BASE [3].

Copyright © 2004 John Wiley & Sons, Ltd.

Whilst these techniques comprise an indispensable toolkit for functional genomics, this kind of analysis fails to address how levels of gene expression relate to the role of gene products in protein interaction networks, particularly in metabolic and signalling pathways, which form the basis of integrated biological processes and cellular behaviour. To address this question, a complementary set of applications is being developed that enable the analysis of microarray data on biological pathways (see e.g. GenMAPP [4], CMAP [2] and KEGG [8]).

Building on these developments, a next generation of functional genomics analysis tools can be envisaged that would integrate identification of differentially expressed genes by statistical techniques, clustering of co-expressed genes and interpretation of gene expression on pathways and networks in a single software environment. The MicroCore project aims to provide a workbench of this kind as an open source platform-independent Java2 toolkit for microarray analysis.

### The MicroCore analysis suite

A suite of analysis programs has been developed for the analysis of gene expression and proteomic data by visualization on cellular pathways. MicroCore is highly extensible and has a pluggable interface for the docking of new and updated software modules, also allowing the inclusion of personalized programs. MicroCore is currently customized for Affymetrix data, including data normalization and annotation via links to external databases, and is adaptable for analysis of data created by other types of microarrays.

Java2 is a powerful object-orientated programming language from Sun Microsystems [14] and has the unique advantage of enabling common source codes to run on almost any platform and operating system. Java2 has an extensive library for graphical user interface representation, as well as support for a wide range of application-related tasks, such as database connectivity, server/client-side and Internet functions, multithreading capability, and even 3D graphics support. This makes Java2 a perfect language for the kinds of applicability one needs to tackle the analysis of microarray data.

The initial release version 1.0 of the MicroCore suite provides two program modules — PIMs (protein interaction maps) and MicroExpress. Together, these two programs enable the user to create user-defined protein interaction maps (Figure 1) and view the differences in gene expression between two microarray experiments in the context of those pathways (Figure 2), making the version 1.0 toolkit already a powerful way to analyse data in parallel with clustering techniques. PIMs and MicroExpress are SBML (Systems Biology Markup Language)-compliant [7], enabling export of pathway maps to other SBML-aware applications.

Additionally, both PIMs and MicroExpress have an inbuilt web browser launcher and Affymetrix Chip annotation tables that allow the user to connect to up to 10 online databases (including NCBI's GenBank, OMIM, LocusLink, Affymetrix NettAffx, TIGR and others). This functionality accommodates most Affymetrix chip types.

Other functionalities include the incorporation of normalization methods (*global normalization*, for Affymetrix arrays, and *global scaling*, which can be applied to both Affymetrix and spotted array data). The normalization methods are calculated *live*, meaning the user can switch to either of the methods or switch them both off and the effect can be seen immediately. Live graphics have been applied to the log function view also, which allows the user to see differential expression after the logarithm of the ratio has been calculated. Statistical methods included in version 1.0 include a Welch's $t$-test for checking the significance of the difference between the data distributions of the two array experiments in question and a $t$-test for checking the significance of differences in expression between experiments for each individual gene.

Ultimately, elucidation of the cellular control of metabolic and signalling networks, and of the significance of genome activation states in these regulatory processes, will require direct comparisons to be made between the transcriptome (measured by microarray technology) and the proteome (measured through proteomic technologies). At present, despite its genome-scale potential, proteome analysis is at a much earlier stage of development than gene expression (microarray) studies [1]. In anticipation of technological hurdles being overcome and proteomic data becoming more widely available, a visualization feature for the display of quantitative proteomic data along with the gene expression data

Copyright © 2004 John Wiley & Sons, Ltd.

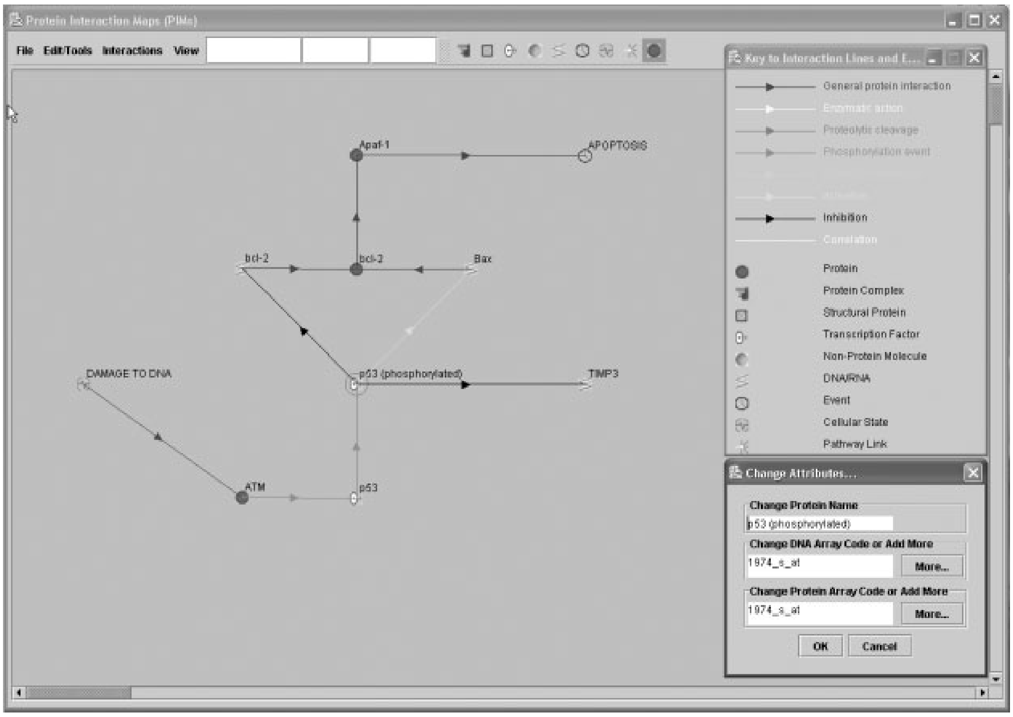

**Figure 1.** MicroCore tools for the creation of user-defined pathway maps enabled for Affymetrix data display

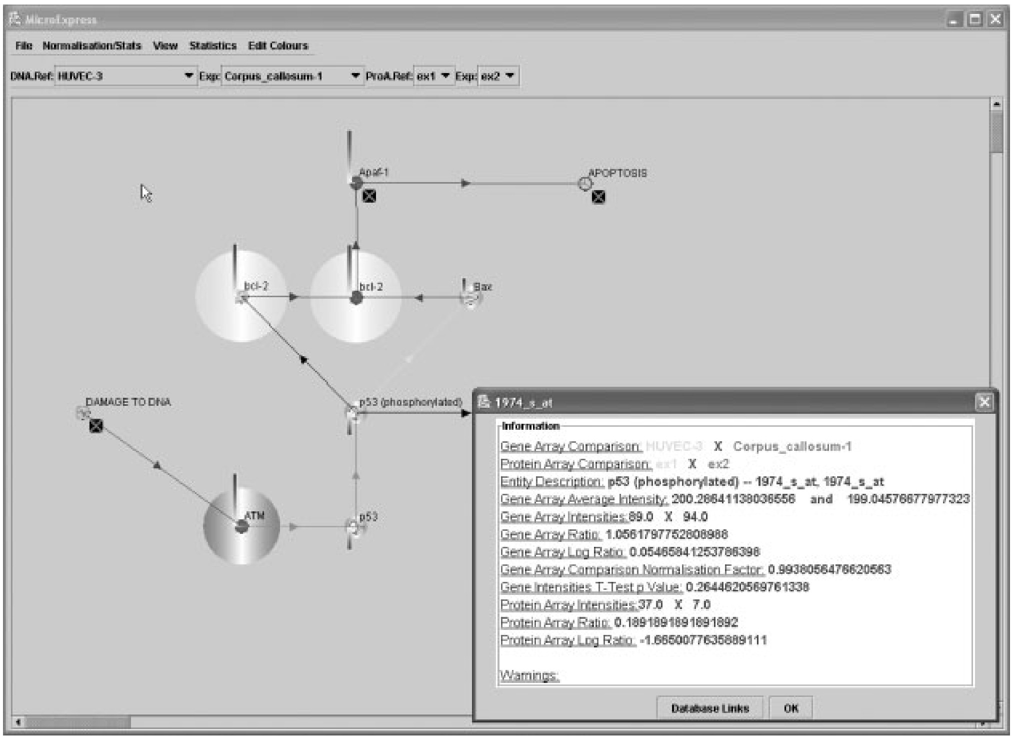

**Figure 2.** Display of differential gene (dark and light grey circle) and protein (dark grey histogram) expression on PIMs maps together with quantitative data has already been included in MicroExpress version 1.0 (Figure 2).

The MicroCore suite is specifically designed for ease of use and, perhaps most importantly, for further integration and expandability. Implementation of fuzzy clustering and statistical modules is in progress for release with version 2.0, which will also include an auto-updating facility. Detailed program documentation is available at the MicroCore website to provide support for users and facilitate development of new application modules. An in-depth tutorial on general microarray analysis methods is also available at this site.

### Acknowledgements

We would like to thank our sponsors, the BBSRC, Microarray analysis summer school, Grant No. BEP17056 (for Microarray Tutorial on website only).

### References

1. Boguski MS, McIntosh MW. 2003. Biomedical informatics for proteomics. *Nature* **422**: 233–237.
2. CMAP; **http://cmap.nci.nih.gov**
3. Dudoit S, Gentleman RC, Quackenbush J. 2003. Open source software for the analysis of microarray data. *Biotechniques* March (suppl): 45–51.
4. Dahlquist KD, Salomonis N, Vranizan K, Lawlor SC, Conklin BR. 2002. GenMAPP, a new tool for viewing and analysing microarray data on biological pathways. *Nature Genet* **31**: 19–20.
5. Eisen MB, Spellman PT, Brown PO, Botstein D. 1998. Cluster analysis and display of genome-wide expression patterns. *Proc Natl Acad Sci USA* **95**: 14 863–14 868.
6. GOLD database; **http://wit.integratedgenomics.com/GOLD/**
7. Hucka M, Finney A, Sauro HM, *et al*. 2003. The systems biology markup language (SBML): a medium for representation and exchange of biochemical network models. *Bioinformatics* **19**: 524–531.
8. KEGG; **http://www.genome.ad.jp/kegg**
9. Lipshutz RJ, Fodor SP, Gingeras TR, Lockhart DJ. 1999. High density synthetic oligonucleotide arrays. *Nature Genet* **21**: (suppl): 20–24.
10. Ross DT, Scherf U, Eisen MB, *et al*. 2000. Systematic variation in gene expression patterns in human cancer cell lines. *Nature Genet* **24**: 227–235.
11. Scherf U, Ross DT, Waltham M, *et al*. 2000. A gene expression database for the molecular pharmacology of cancer. *Nature Genet* **24**: 236–244.
12. Slonim DK. 2002. From patterns to pathways: gene expression data analysis comes of age. *Nature Genet* **32**: (suppl): 502–508.
13. Sturn A, Quackenbush J, Trajnoski Z. 2002. Genesis: cluster analysis of microarray data. *Bioinformatics* **18**: 207–208.
14. Sun Website — **http://java.sun.com**
15. Valafar F. 2002. Pattern recognition techniques in microarray data analysis: a survey. *Ann N Y Acad Sci* **980**: 41–64.

Copyright © 2004 John Wiley & Sons, Ltd. 
